# A Novel Approach for Lie Detection Based on F-Score and Extreme Learning Machine

**DOI:** 10.1371/journal.pone.0064704

**Published:** 2013-06-03

**Authors:** Junfeng Gao, Zhao Wang, Yong Yang, Wenjia Zhang, Chunyi Tao, Jinan Guan, Nini Rao

**Affiliations:** 1 College of Biomedical Engineering, South-Central University for Nationalities, Wuhan, People’s Republic of China; 2 Department of Biomedical Engineering, Case Western Reserve University, Cleveland, Ohio, United States of America; 3 School of Information Technology, Jiangxi University of Finance and Economics, Nanchang, People’s Republic of China; 4 School of Life Science and Technology, University of Electronic Science and Technology of China, Chengdu, People’s Republic of China; CSIC-Univ Miguel Hernandez, Spain

## Abstract

A new machine learning method referred to as F-score_ELM was proposed to classify the lying and truth-telling using the electroencephalogram (EEG) signals from 28 guilty and innocent subjects. Thirty-one features were extracted from the probe responses from these subjects. Then, a recently-developed classifier called extreme learning machine (ELM) was combined with F-score, a simple but effective feature selection method, to jointly optimize the number of the hidden nodes of ELM and the feature subset by a grid-searching training procedure. The method was compared to two classification models combining principal component analysis with back-propagation network and support vector machine classifiers. We thoroughly assessed the performance of these classification models including the training and testing time, sensitivity and specificity from the training and testing sets, as well as network size. The experimental results showed that the number of the hidden nodes can be effectively optimized by the proposed method. Also, F-score_ELM obtained the best classification accuracy and required the shortest training and testing time.

## Introduction

Deception is an important social and legal behavior. The traditional method for detecting deception is based on polygraph measurements. In recent years, significant progress in neuroscience has inspired investigations on lie detection. A number of studies have used neurophysiological signals, such as Functional Magnetic Resonance Imaging (fMRI) and Event Related Potential (ERP) [1]–[3], to investigate lie detection [4]. An endogenous ERP component, P300 (P3), has been extensively investigated and successfully used in the detection of deception and malingering [5]–[7].

The widely used P3-based lie-detection methods can be roughly divided into three categories: bootstrapped amplitude difference (BAD), bootstrapped correlation difference (BCD) [8] and pattern recognition (PR) methods [4], [9], [10]. Compared with BAD and BCD, PR-based lie detection is a promising approach for two main reasons: 1) more physiological features can be extracted from raw P300 and 2) a variety of PR classifiers can be utilized to improve the accuracy of the lie detection. However, the adoption of PR classifiers for lie detection has not yet been widely reported. Davatzikos et al. [4] proposed a support vector machine (SVM)-based method to classify the patterns of brain activity (fMRI data) obtained during lying and truth-telling. Abootalebi et al. [9] used linear discrimination analysis (LDA) to identify P3 responses and obtained a higher detection rate (86%) than that obtained using BAD- and BCD-based methods. SVM was used for the first time in the investigation of P3-based lie detection by Gao et al. [10]. Compared to fisher discrimination analysis (FDA) and back-propagation neural networks (BPNN), SVM classifier obtained the highest average classification accuracy (91.8%) between P3 responses from the guilty and non-P3 responses from the innocent.

In the current studies of EEG classification, there is a general trend to test various classifiers to ultimately obtain the highest classification accuracy possible [11], [12]. Rooted in statistical learning theory, SVM classifier implements structural risk minimization and margin hyperplane maximization [13]. More importantly, the SVM can map the nonlinear separable data onto a high-dimension space, and hence classify the data linearly by using a technique of kernel function mapping. In the past 30 years, SVM classifier has demonstrated great advantages over most other classifiers in terms of classification accuracy and generalization power [4], [11], [14]. However, it should be noted that the time required to train the classification models should be considered, especially when the training data is substantial and the training procedure is complex. Taking this into account, SVM and gradient descent-based artificial neural network (ANN, e.g., BPNN) may be unsuitable and unsatisfactory due to their high computational cost [15], [16]. Extreme learning machine (ELM), a single-layer feedforward network (SLFN)-based method, was proposed by Huang et al. [15] to overcome some inherent drawbacks of SVM and BPNN (complex and long parameter training procedure). ELM randomly specifies the input weights and biases and then analytically calculates the output weights with the smallest norm. Hence, ELM tends to provide good generalization power at an extremely fast training speed [17], [18]. During the past several years, ELM has drawn considerable attentions in many fields related to PR [19], [20].

Some researchers have studied the performance of ELM in the classification of ERP. Liang et al. applied ELM for the first time to the classification of mental tasks using EEG signals [21]. Their results showed that ELM obtained similar classification accuracy with a training time that was 1–2 orders of magnitude shorter, compared with SVM and BPNN. ELM was successfully adopted by Shi et al. for EEG-based vigilance estimation [22]. Several ELM-based investigations on epileptic seizure detection have also demonstrated the promising performance of ELM in the classification of different EEG tasks [16], [23], [24]. To date, ELM has not been used to detect lying and to classify guilty and innocent subjects.

Feature selection plays an important role in the construction of a classification model. Chen demonstrated for the first time that feature selection strategies for SVM classification should be included [25]. Polat et al. classified medical datasets using a hybrid system of feature selection and several classifiers and obtained better performance compared with the methods that did not utilize feature selection [26]. Akay proposed a breast cancer diagnosis method which integrated SVM and F-score feature selection [27]. The experimental results showed that the hybrid method attained the higher classification accuracy compared with all other models without feature selection. To date, few researchers have conducted studies combining ELM with feature selection. Han et al. combined principal component analysis (PCA) and ELM to predict the postoperative survival time of patients who suffered from non-small cell lung cancer [24]. Their results showed that the CPU time with their proposed method was significantly less than that obtained with other classification models, such as BPNN and BPNN combined with PCA. In the area of EEG classification, no reported investigation has combined ELM with feature selection.

The number of hidden nodes (*NHN*) in ELM is an important parameter that may affect the classification performance (the other one important parameter the activation function). *NHN* is usually randomly assigned in the basic ELM algorithm. Huang et al. found that for some special datasets, the generalization performance of ELM was very stable over a wide range of *NHN* [15]. However, Cao et al. indicated that the classification boundary may not be optimal when this number remains unchanged during the training procedure [28]. In addition, too many or too few *NHN* might lead to over-fitting or under-fitting [22]. Because this is one of the hottest issues related to the ELM research, a few methods were recently proposed to investigate this problem [29]–[33]. However, these improved ELM algorithms are relatively complicated for real application in classification system. Moreover, similar to SVM algorithm, ELM cannot directly obtain the feature importance. Finally, there exists a close relationship between the *NHN* and the dimensions of the feature space, which, however, was not stressed in these improved algorithms.

In this study, we combined ELM with feature selection to classify truth-telling and lying signals. In addition, we simultaneously optimized the feature subspace and the *NHN* in ELM. We hypothesize that this joint optimization strategy could not only further enhance the classification accuracy of lie detection, but also significantly decrease the training and testing times.

## Materials

### 1. Ethics Statement

The experiment was approved by Psychology Research Ethical Committee (PREC) of the College of Biomedical Engineering in South-Central University for Nationalities. Thirty-three healthy subjects (15 females, mean age of 22) were recruited from the university. The participants provided their written informed consent according to a human research protocol in this study.

### 2. Subjects and Experimental Protocol

The guilty knowledge test (GKT) [9] and three-stimulus protocol [10] were used in this study. The probe (P) stimuli consisted of some images or sound related to criminal acts, such as the weapon in the scene of the crime. The guilty is certainly familiar with these stimuli, whereas this is not the case for the innocent. The target (T) stimuli are known by all the subjects, but these are not related to the criminal acts. The irrelevant (I) stimuli are not known by all the subjects and are not related to criminal acts. All of the participants were randomly divided into a guilty group and an innocent group. Six different jewels were prepared, and their pictures served as the stimuli during the detection procedure. A safe containing one (for the innocent) or two (for the guilty) jewels was given to each subjects, who were told that only one examiner knew the contents in the safe. The subjects were instructed to open the safe and memorize the details of the object. All of the subjects were asked to write down the information of the objects in the safe, such as styles and colors. As the subjects stole the jewels, all of the researchers were asked to stay out.

We instructed the guilty steal one jewel and pocket the object, which served as a P stimulus, whereas the other one in the safe served as T stimulus and the remaining four pictures were I stimuli. The guilty group was instructed to press the “Yes” and “No” buttons when facing with T and I stimuli, respectively. With a P stimulus, they were asked to press the “No” button in an attempt to hide the stealing act. We told the guilty that they would earn 100 RMB if successfully concealed the identity of the probe stimuli during the experimental session. For the innocent, the object in the safe was a T stimulus, whereas the object stolen by guilty subjects servered as a P stimulus in order to add comparability (although the object is indeed random in the remaining five pictures); the other four images were I stimuli. In contrast, the innocent group responded honestly to all of the stimuli.

### 3. EEG Data Acquisition

All of the subjects were seated in a chair, facing a video screen 1 m from their eyes. The stimuli were presented in a random order on the screen for a duration of 0.5 s at a random interval of 1.4–1.6 s. Each session lasted approximately 5 minutes with a 3-minute resting time. Each subject was instructed to participate in 3 sessions. The EEG was recorded on the following nine silver electrodes: C3, Cz, C4, P3, Pz, P4, O1, O2, and Oz from an International 10–20 system. The vertical EOG (VEOG) signals and the horizontal EOG (HEOG) signals were recorded. The EEG and EOG signals were passed through a Neuroscan Synamps Amplifier with a bandpass filter of 0.1–30 Hz, and digitized at 500 Hz. All of the electrodes were referenced to the right earlobe, and the electrode impedances were less than 2 kΩ. The artifact removal criterion was 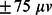. The EEG data obtained from *5* of the subjects were excluded due to significant eye blinking and eye movement artifacts. Although none did, any subjects with a clicking error rate of more than 5% would be excluded. Finally, EEG signals from 14 subjects in each group were further preprocessed.

### 4. Preprocessing

During the experiment, if the subject did not provide a response or failed to give the right response by pressing the corresponding button, the corresponding EEG responses were first rejected by visual inspection. The resulting EEG data were then segmented into epoched datasets from 0.2 s before to 0.8 s after the stimuli onset. All of the trials were baseline-corrected based on the pre-stimulus interval.

**Figure 1:**
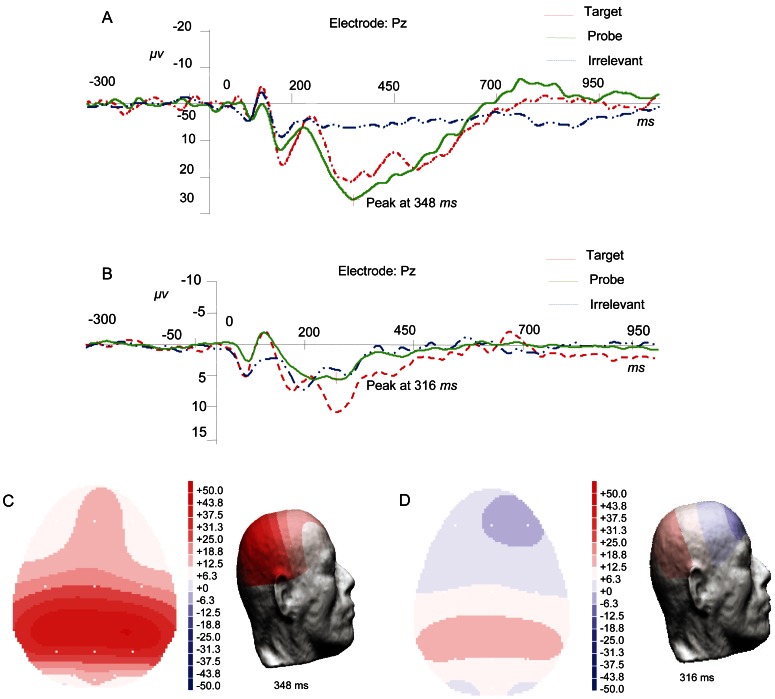
The preprocessing results of a guilty subject and an innocent subject. 1A: Three averaged waves over the three kinds of stimuli respectively at Pz electrode from the guilty subject. 1B: The averaged waves over the three kinds of stimuli respectively at Pz electrode from the innocent subject. 1C: The brain topographies at the latency of 348 ms of the averaged P responses (the solid line in Figure 1A). 1D: The brain topographies at the peak point of 316 ms of the averaged P responses (the solid line in Figure 1B).

All of the epoched datasets were further processed as follows. Within each subject, 30 single-trials from each type of stimulus and each site were pooled into one wave. Figure 1 shows the results from a randomly selected guilty subject and an innocent subject. The averaged waves at the Pz electrode from the guilty and innocent subjects are shown in Figure 1A and Figure 1B, respectively. Comparing the two subfigures, we can observe that there is significant P300 in the P responses (i.e., the response waves from the P stimuli) from the guilty subject, but no P300 in the P responses from the innocent subject. Furthermore, the brain topographies at the latency of 348 ms (see Figure 1A) and at the peak point of 316 ms (see Figure 1B) are shown in Figure 1C and Figure 1D, respectively. By comparing the two figures, we can observe that there exists a significant difference between the P responses on the Pz site from the two subjects. Similar to some early reports [34], [35], all of the P responses on the Pz site were finally selected, and each of the 5 P responses for each subject were pooled into one average to enhance the signal-to-noise ratio (SNR) of the P300 [10], [36]. Hence, there were approximately 300 P responses for each group of subjects. The P responses from the guilty subjects represent P3, whereas the responses from the innocent subjects represent non-P3.

## Methods

### 1. Feature Extraction

Three groups of features based on time-domain, frequency-domain, and time-frequency domain features were extracted from each P response with the time varying from 0.2 to 1 s. Burg’s method was used for spectrum estimation [14]. There were nine time- and frequency-domain features as follows: maximum amplitude *V*_max_, latency *t*_max_, latency/amplitude ratio *R*_L/A_, minimum amplitude *V*_min_, peak-to-peak amplitude *V*_ptp_, positive area *A*_p_, maximum frequency *f*_max_, mean frequency *f*_mean_, and the power of the frequency band containing the P3 *A*_lf_. In this study, we used discrete wavelet transform (DWT) [11], [37] to decompose each P response into seven sets of wavelet coefficients. The coefficient set corresponding to the first frequency band (0.1 to 3.9 Hz) was selected as the 22 wavelet features, which were denoted by *W* where *i = *1, 2,..., 22. Please refer to our previous report for more details on the extracted features [36]. After the feature extraction, two feature sample sets (represent P3 and non-P3) were obtained with the class label 1 and −1, respectively. Each sample consisted of 31 feature values. Before the classification, all of the feature values were normalized to [−1, 1].

### 2. Feature Selection

The feature selection can help the original classification system achieve a better predictive performance and a lower computational cost by removing any redundant features. The F-score is a simple but effective technique for evaluating the discriminative power of each feature in the feature set. Chen proposed and combined this method with SVM to participate NIPS 2003 Feature Selection Challenge and was ranked third [38]. Recently, many researchers have successfully applied the combination of F-score with an SVM classifier to various classification tasks [25], [26], [27], [39].

Given the *i*th feature vector 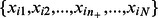 with the number of positive instances 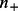 and the number of all the instances *N*, the *F-score* value of the *i*th feature is defined by
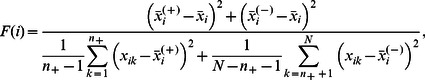
(1)where 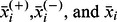 are the average of the positive, negative, and whole samples, respectively, and 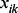 is the *k*th feature value in the *i*th feature vector. The numerator indicates the discrimination between the positive and the negative sets, and the denominator is the sum of the deviation within each feature set. A larger the *F-score* value indicates that the feature has more discriminative power. We adopted the F-score method in this study due to its simplicity of its use in a lie detection system with real applications.

There are two main methods that are used to select the appropriate feature subset: the filter method [40] and the wrapper method [41], [42]. Although there is higher computation cost associated with the wrapped method, many experimental results are in favor of the wrapper method for feature selection due to its good performance. Hence, we also used this method in this study.

For comparison, we adopted another popular method, principal component analysis (PCA), to select the features [43]. PCA extracts dominant features from the original input samples. The dominant features retain most of the information, both in the sense of maximum variance of the features and in the sense of minimum reconstruction error [44]. In this study, similarly to the F-score, PCA was combined with classifiers to identify the optimal feature set.

Given a set of *N* input samples 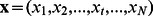, each of which has *m* dimensions 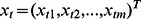, PCA first solves an eigenvalue problem, i.e.,
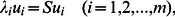
(2)where 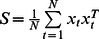 is the sample covariance matrix,and 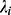 is the corresponding eigenvalue of the eigenvector 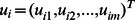. After all of the 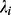 are sorted in descending order, PCA uses the first *d* eigenvalues and their corresponding eigenvectors to project the original input samples 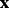 into a *d*-dimensional space using the following linearly transform:
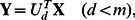
(3)where 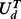 is a 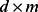 matrix, the *i*th row of which is the eigenvector 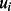. Each feature vector of the new projection samples **Y** is referred to as a principal component.

### 3. Extreme Learning Machine

For comparison purposes, ELM, SVM, and BPNN were selected as the three types of representative machine learning-based classifiers to classify the P300 data for lie detection.

**Figure 2:**
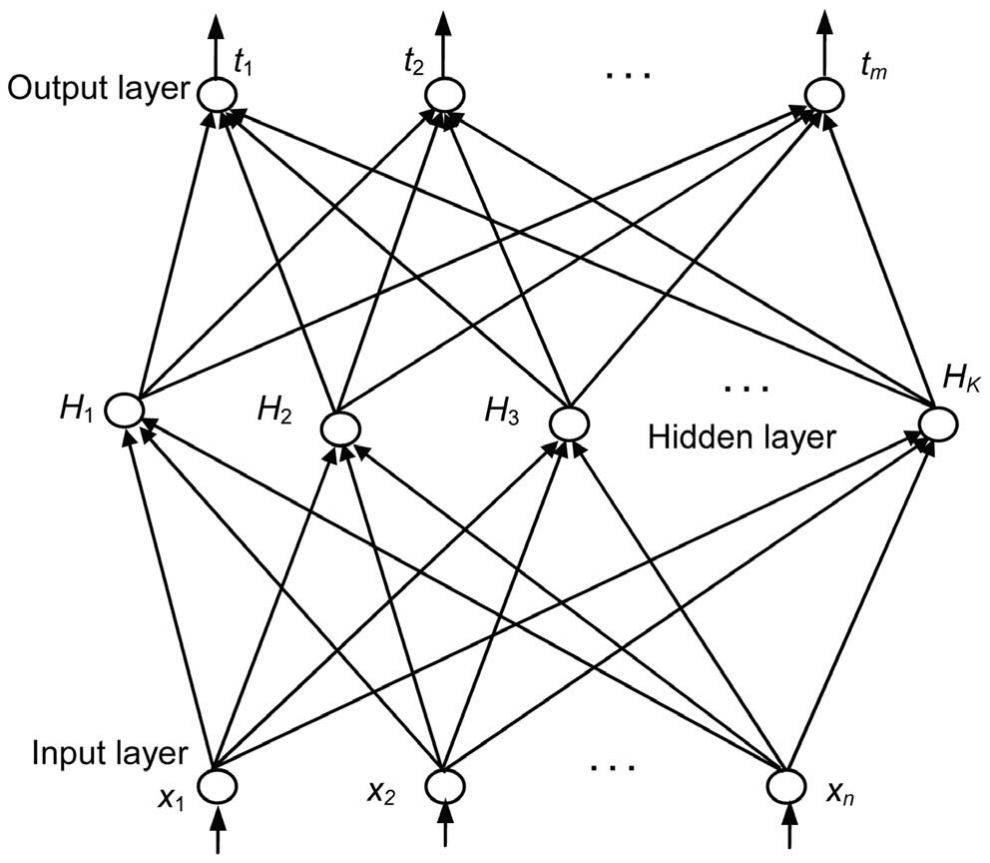
SLFN with *K* hidden, *n* input and *m* output nodes.

Given *N* different training instances 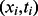, where 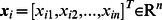 and 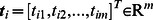, we train a SLFN with *K* hidden nodes and an activation function 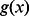, as shown in Figure 2. This network can be mathematically modeled as
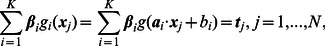
(4)where 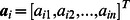 denotes the weight vector connecting the *i*th hidden node and the *n* input nodes, 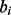 is the bias of the *i*th hidden node, 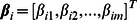 denotes the weight vector connecting the *i*th hidden node and the *m* output nodes, and 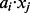 denotes the inner product of 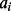 and 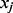.

The above *N* equations can be rewritten in a matrix form as
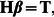
(5)where



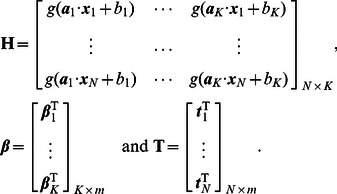
(6)
**H** is called the hidden-layer output matrix, where the *i*th column is the output of the *i*th hidden node [26]. To learn the *N* instances for a SLFN, the conventional method is to find the solution set 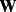, including 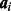, 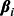 and 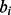, by minimizing the following cost function:
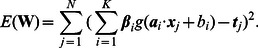
(7)


Given an arbitrarily small value 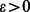, Huang et al. proved that if the input weights and the biases of the hidden nodes are randomly assigned and the activation function in the SLFN is infinitely differentiable, the SLFN can approximate the *N* training data with 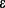 error, i.e., 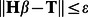 [15]. In this case, the matrix **H** has been randomly fixed. Hence, the training procedure of SLFN is equivalent to the identification of a least-squares (LS) solution of the linear system:
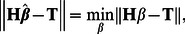
(8)where 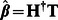 is the LS solution of the above problem with the smallest norm, and 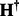 is the Moore-Penrose generalized inverse of **H**. Bartlett [17] and Huang et al. [15] indicated that SLFNs with smaller output weights have a better generalization ability.

### 4. The Proposed Method: F-score_ELM

In this study, we combined the ELM methodology with a feature selection method for lie detection. There are two important problems for the proposed method: the choice of the optimal feature subset for F-score and the determination of the value of *NHN* for ELM.

Taking into account a lie diction system with real applications, the wrapper method mentioned previously should be more suitable for solving the first problem than the filter method because the feature subset was relatively fixed after the training procedure. With respect to the optimal *NHN*, we did not randomly assign but integrated the optimization of *NHN* into the selection of feature subset. The proposed method is referred to as F-score_ELM.

**Figure 3:**
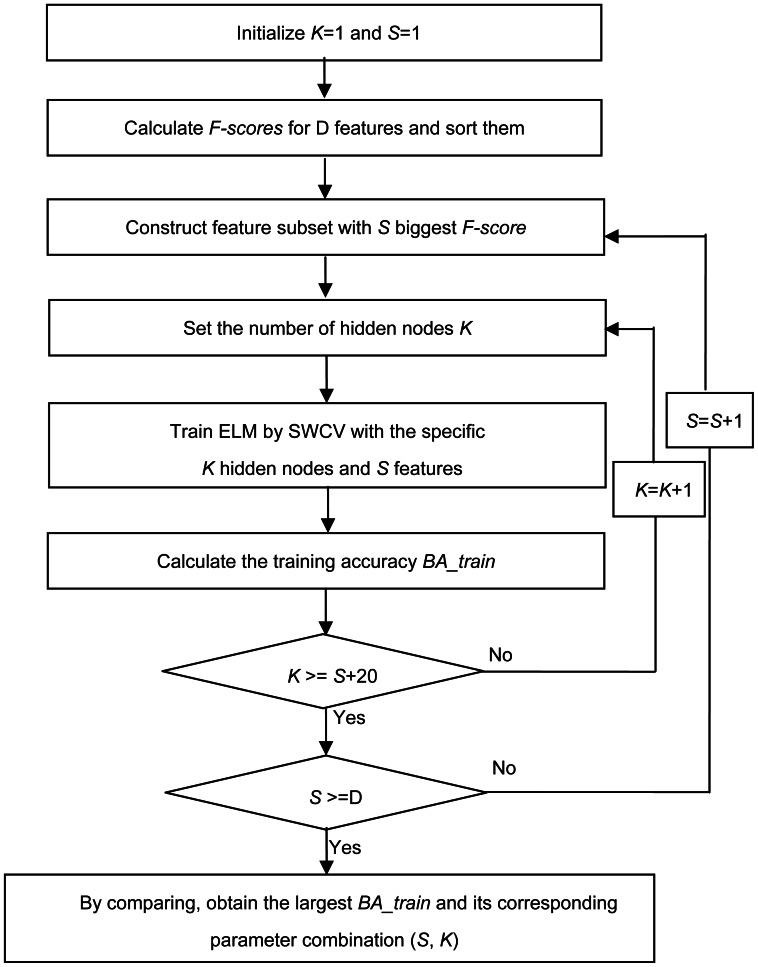
The block diagram of the proposed method F-score_ELM.

Figure 3 presents the block diagram of F-score_ELM using a grid-search technique [45] to jointly optimize the feature subset and the *NHN* in ELM. Let *D* denote the number of the originally extracted features, which equals 31 in this paper. The F-score_ELM method consists of the following steps:

*Step 1*: Calculate the *F-score* values of the *D* feature vectors. Then, rearrange the feature vector set such that the first feature vector has the highest *F-score*, and the second vector has the second highest *F-score*, and so on. Let **F** denote the new feature vector set, and initialize a feature subset, denoted by **FS**, to be empty.

*Step 2*: Pick one feature vector with the highest *F-score* value from **F**. Add the selected vector to the subset **FS**. Set *S* to be the number of features in subset **FS**.

*Step 3*: Denote the *NHN* of the ELM by *K* and initialize *K* = *S*.

*Step 4*: Feed subset **FS** into the ELM classifier with *K* hidden nodes to train and search for the optimal combination (*S*, *K*). Considering the specific requirements for lie detection and to avoid over-fitting problem, a Subject-Wise cross validation (SWCV) [46] was adopted, resulting in 14 pairs of training sets and testing sets. Furthermore, a 10-fold CV was performed on each pair of training set. Hence, the averaged accuracy, denoted by *BA_train*, is calculated by averaging the values of 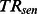 (the *mean* of 14 sensitivities [47]) and 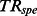 (the *mean* of 14 specificities).

*Step 5*: Update *K* to *K+*1. Repeat *Step 3* though *5* until *K* = *S+20* (based on prior knowledge and the computational limitation of the lie detection system), as shown by the inner loop in Figure 3.

*Step 6*: Update *S* by *S+*1. Repeat *Step 2* through *6* until **F** is empty, as shown by the outer loop in Figure 3.

*Step 7*: Comparing all of the *BA_train* values obtained in *step 4*, the optimal parameter combination (*S*, *K*) is finally obtained when the *BA_train* reaches its highest value. Accordingly, the solution 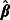 and its corresponding value of the hidden node 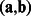 in the ELM are also obtained, which are fixed and then used in testing phase.

*Step 8*: Calculate the testing accuracy on the 14 pairs of testing sets with the optimal feature subset and the trained ELM. Hence, 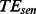 (the *mean* of the 14 sensitivities) and 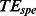 (the *mean* of the 14 specificities) can be obtained.

In the *Step 4* and *Step* 8, the sensitivity and the specificity refer to the percentage of the correctly classified feature samples with the class label 1 (P3 class) and −1 (*non*-P3 class), respectively.

To objectively evaluate the performance of the proposed method, the following combined classification models were also performed: PCA_ELM, PCA_BPNN, PCA_SVM, F-score_BPNN and F-score_SVM. Three individual classification models (i.e., the models without integrating feature selection) were also conducted: ELM, BPNN and SVM. Each individual model was trained only to obtain the optimal classifier parameters when the training accuracy *BA_train* reached its highest value.

For the models that utilized PCA, the eigenvalues were first calculated and sorted in a descending order. Then, the transformed new feature set was constructed using the *d* largest eigenvalues. The new feature set was then fed into the classifiers. Similar to F-score_ELM, we used grid-search technique to jointly optimize the optimal value of *d* (see Section Feature Selection) and the classifier parameters.

In this study, a sigmoid activation function 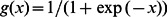 was used in all of the classification models to fairly and objectively compare these models. The learning rate 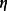 and the control precision 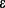 of the models that integrated BPNN were set to be 0.025 and 0.002, respectively; The Levenberg-Marquardt algorithm was used for the training of these models, and the *NHN* of BPNN was also optimized by the grid-searching. The training and testing strategies mentioned above were also used for the models that utilized the SVM, and, based on our previous experience, the penalty parameter *C* and the radial width 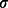 for radial basis function (RBF) [48] (kernel function 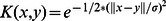) were tuned with the following grid: *C* = [2^5^,..., 2^8^], 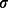 =  [2^3^,..., 2^6^] (step size = 2^1^ ). To decrease the huge training time, 10-fold SWCV and then normal 5-fold CV, which consists of a three-dimensional grid-search procedure, were used in the training stage for BPNN and SVM.

Using the optimization procedure described above, the following measures were used to evaluate the performance of the total nine classification models:

- The training accuracy. This measure consists of the sensitivity, the specificity, and their respective standard deviations (SDs). They correspond to 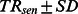 and 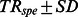 when the corresponding *BA_train* reaches its highest value.
- The test accuracy. Similar to the above measures, This measure refers to 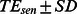 and 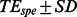. Additionally, all of the 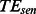 and 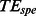 are averaged to obtain a balanced testing accuracy, which is denoted by *BA_test*.
- The optimal number of features in the feature subset when the classification model reaches the highest value of *BA_train*. This optimal number is denoted by *NFS*.
- The optimal classifier parameters when the classification model reaches the highest valus of *BA_train*. For the models that integrate BPNN and ELM, this parameter is the optimal value of *NHN*, whereas for the models that integrate SVM, the number of the support vectors (*NSV*) is used to compare the models with ELM and BPNN.
- The training time of the classification models, which refers to the time spent on the *Step 1* through and is denoted by *TTR*.
- The testing time of the classification models, which refers to the time spent on testing the 14 pairs of unseen testing sets. For individual models, which is denoted by *TTE*, refers to the time required to test the 14 pairs of unseen datasets in the original feature space.

## Results

Table 1 shows the *F-score* values of the 31 original features. Those features with relatively larger *F-score* values were selected to construct the feature subset. The detailed results of the above mentioned six measures are summarized in Table 2. Furthermore, we listed the values of *BA_train* and *BA_test* in Table 3 for each classification model. Finally, the averaged value of each pair of the sensitivity and the specificity in each model was calculated for training and testing, respectively, which yielded 14 balanced accuracies forthe training (and the testing) for each model. Hence, the paired *t*-test was performed between F-score_ELM and each of the other models to obtain the corresponding significance level (*p* value) of the difference of the balanced accuracy. These significance levels are also provided in Table 3.

**Table 1:** The results of feature valuation on original 31 features using F-score method.

Features	*F-score* values
*V*_max_	**0.966**
*t*_max_	0.581
*R*_L/A_	0.162
*V*_min_	0.019
*V*_ptp_	**0.890**
*A*_p_	0.124
*f*_max_	0.001
*f*_mean_	0.305
*A*_lf_	**0.886**
*W*_1_– *W*_5_	0.075, 0.001, 0.317, 0.002, 0.075,
*W*_6_– *W*_10_	0.003, 0.154, 0.097, 0.069, 0.364,
*W*_11_–*W*_16_	**0.977**, 0.554, 0.213, **0.886**, 0.874, **0.987**
*W*_17_–*W*_22_	**0.893**, **0.953**, **0.892**, **0.987**, 0.874, **0.881**

**Table 2:** Performance of the classification models with the optimal *NFS* and *NHN* (or *NSV*).

Classification models	*Times*		*Accuracy (%)*				*NHN/NSV*	*NFS*
	*TTR(h)*	*TTE(s)*	Training		Testing			
			*TR_sen±SD_*	*TR_spe±SD_*	*TE_sen±SD_*	*TE_spe±SD_*		
**BPNN**	1.25	0.51	95.31±1.38	88.61±4.44	95.05±3.25	86.38±3.15	45	31
**SVM**	20.22	22.06	97.88±0.41	97.68±0.41	96.33±1.87	95.15±2.29	58.28	31
**ELM**	0.03	0.004	98.72±0.35	98.16±0.51	98.26±0.32	98.14±0.44	51	31
**PCA_BPNN**	106.64	1.59	95.10±1.82	95.75±1.97	98.20±1.82	98.34±1.93	20	14
**PCA_SVM**	675.58	25.23	99.32±0.35	99.28±0.33	99.65±0.02	98.91±0.02	57.76	14
**PCA_ELM**	0.64	0.014	98.82±0.36	99.31±0.36	98.99±0.48	98.79±0.51	30	13
**F-score_BPNN**	58.58	0.184	95.26±0.23	88.05±1.36	95.80±3.92	90.05±5.27	34	25
**F-score_SVM**	633.77	21.62	98.06±0.48	98.21±0.44	97.68±0.02	97.95±0.02	60.25	25
**F-score_ELM**	0.61	0.003	99.42±0.38	98.52±0.72	99.27±0.24	98.17±0.25	29	11

**Table 3:** Balanced accuracy of each model and statistical analysis results between F-score_ELM and the other models.

Classification models	*Balanced accuracy and statistical results (%)*			
	Training		Testing	
	*BA_train*	*p value* (2-t)	*BA_test*	*p value* (2-t)
**BPNN**	91.96	0.000*	90.72	0.000*
**SVM**	97.78	0.000*	95.74	0.000*
**ELM**	98.44	0.044^▴^	98.20	0.008*
**PCA_BPNN**	95.43	0.000*	98.27	0.009*
**PCA_SVM**	99.30	0.006*	99.30	0.004*
**PCA_ELM**	99.06	0.233	98.89	0.173
**F-score_BPNN**	91.66	0.000*	92.93	0.000*
**F-score_SVM**	98.14.	0.0008*	97.82	0.006*
**F-score_ELM**	98.97	[]	98.72	[]

“*” and “^▴^” denotes *p* value<0.01 and *p* value<0.05, respectively for the comparison of the F-score and the indicated model using paired *t*-test.

### 1. General Classification Performance

First, the comparison of the accuracy results in the first three rows in Table 2 shows that ELM, which exhibited training sensitivity of 98.72% and training specificity of 98.16%, performs significantly better than SVM (paired *t*-test, *p*<0.001) and BPNN (paired *t*-test, *p*<0.001). The results of the comparison of the generalization performance are the same as the training results (paired *t*-test, *p*<0.001).

As shown in Table 3, the comparison of the accuracy between the hybrid and its corresponding individual model revealed that each hybrid model achieves significantly higher accuracy than the corresponding individual model, with the exception of F-score_BPNN. For example, the BPNN obtained a *BA_test* value of 90.72%, whereas PCA_BPNN achieved 98.27% (paired *t*-test, *p*<0.0001). Both the *BA_test* and the *BA_train* of F-score_ELM are significantly higher than the corresponding values obtained with the ELM (see Table 3, *p = *0.044<0.05 and *p = *0.008<0.01, respectively).

Second, as shown in the last column of Table 2, it is obvious that the feature selection reduces the value of the *NFS* for the hybrid models from the number of the original features (*NFS* = 31). For example, F-score_ELM selected 11 features (*NFS* = 11), which are most informative to the classification and thus highlighted in Table 1, to construct the feature subset.

Third, the feature selection effectively helped the hybrid models obtain lower values of the *NHN* than the corresponding individual models, as can be observed from the table. For example, *NHN* is equal to 29 and 51 for F-score_ELM and ELM, respectively. Additionally, *NHN* values of 20 and 45 were obtained for PCA_BPNN and BPNN, respectively.

The smaller *NFS* and *NHN* and the higher accuracy obtained with the hybrid models confirms the hypothesis that the combination of the feature selection method and these classifiers improves the classification performance. The above results are analyzed further in the next section.

Comparing the PCA and F-score methods, we can observe that each classifier combined with F-score achieves an accuracy that is similar to that obtained by the same classifier combined with PCA. As shown in Table 3, there is no significant difference of accuracy between F-score_ELM and PCA_ELM (*p = *0.233>0.01 for training and *p = *0.173>0.01 for testing). The results appear to indicate that both F-score and PCA can be successfully combined with these three classifiers to jointly optimize the feature space and the classification parameters for lie detection. However, each classifier combined with F-score requires significantly less computation time than the same classifier combined with PCA. For instance, the *TTR* of F-score_BPNN equals 58.58 hours, whereas the *TTR* of PCA_BPNN equals 106.64 hours. We will compare these two methods further in Discussion.

Moreover, as shown in Table 2, the difference between the *TTR* of F-score_ELM and that of F-score_SVM is almost 1108-fold. In addition, the *TTE* of F-score_ELM is approximately 0.003 s, which is the shortest among all nine models tested. This short time for F-score_ELM should be attributed to the optimized results: the smallest *NHN* (*NHN* = 29) and smallest feature number (*NFS = *11).

### 2. Impact of Network Size and Feature Selection on the Classification Performance

**Figure 4:**
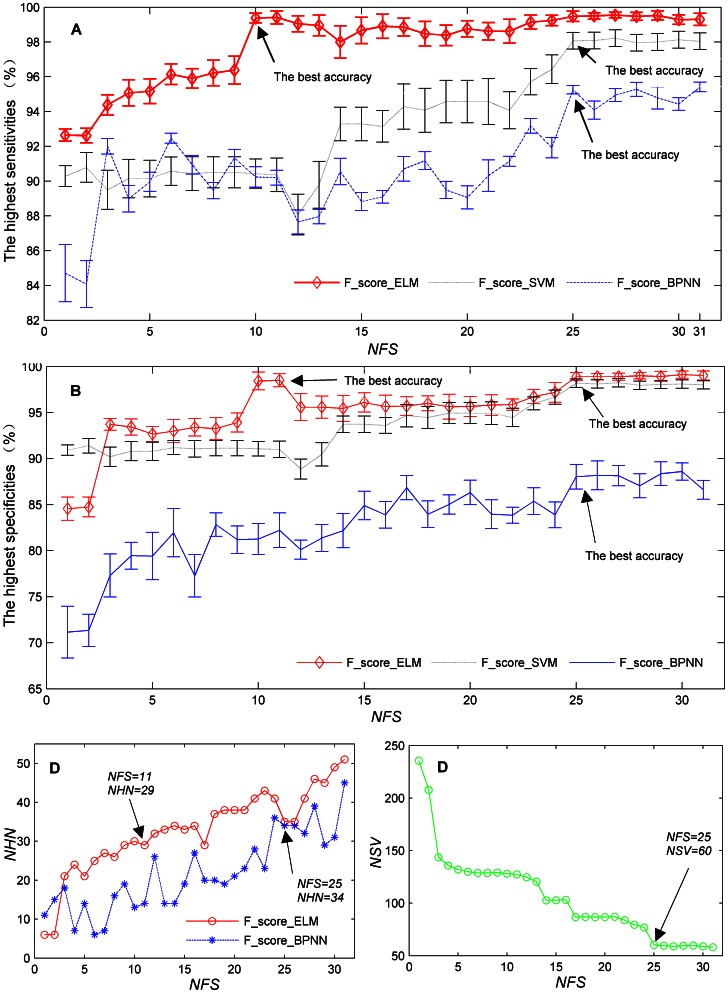
Training accuracy and *NHN*/*NSV* as a function of *NFS* achieved by the three classification models. Each point in each curve corresponds to the highest classification performance of the indicated model with the optimal *NHN*/*NSV*. 4A: Highest sensitivity with the optimal *NHN* or *NSV* vs *NFS*. 4B: Highest specificity with the optimal *NHN* or *NSV* vs *NFS*. 4C: *NHN* vs *NFS* for which *BA_train* achieves its highest value. 4D: *NSV* vs *NFS* for which *BA_train* achieves its highest value.

Based on the above results, we next investigated the effect of the feature selection on the optimal network size and the classification accuracy. In this study, the network size refers to the *NHN* for the ELM classifier and the *NSV* for the SVM classifier. The results of analysis are shown in Figure 4. Each curve in Figure 4A illustrates the highest sensitivities 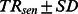 that the indicated classification model can achieve with *NFS* varying from 1 to 31. The specificities 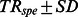 are similarly plotted in Figure 4B. For each model, Figure 4C demonstrates the relationship between the *NFS* and the *NHN* for which *BA_train* achieves its highest value. Similarly, the relationship between the *NFS* and the *NSV* is shown in Figure 4D.

As shown in Figure 4A and 4B, in general, there is no obvious monotonically increasing tendency as the *NFS* increases from 1 to 31. For example, the results of F-score_ELM show a 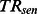 of 99.42% and a 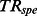 of 98.52% when *NFS = *11; these values are slightly less than the highest corresponding values (99.47% and 98.89%, respectively), which are obtained for *NFS* equal to 25. As shown in Figure 4, this phenomenon is also exhibited by F-score_BPNN and F-score_SVM models. Hence, the accuracy that approximates the highest value with a significantly smaller *NHN* and *NFS* is regarded as *the best accuracy* (i.e., the highest value was not always the optimal for our training purpose). These best accuracies and the corresponding *NHN* and *NSV* for the three models, which correspond to the results in the last three rows in Table 2, are labeled in Figure 4A through 4D.

**Figure 5:**
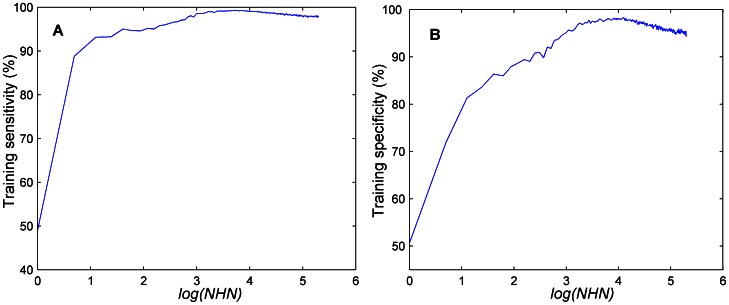
Training accuracy (constant *NFS = *11) as a function of *NHN* achieve by the F-score_ELM. 5A: Highest sensitivity 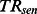 vs *log (NHN).* 5B: Highest specificity 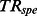 vs *log (NHN).*

We also investigated the individual influence of the *NHN* on the classification accuracy of the ELM classifier. We set the *NFS* to be 11 (the optimal values mentioned previously) and then trained F-score_ELM with a grid search of the *NHN*, which varied from 1 to 200. Figure 5 shows the training accuracy as a function of the *NHN*. Figures 5A and 5B show the sensitivities 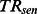 and the specificities 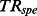, respectively. There is a large fluctuation in the classification accuracy as the *NHN* increases gradually. For example, the sensitivity and specificity only equal 88.8% and 71.7%, respectively when *NHN* is set to be 2, whereas these are 96.03% and 91.02%, respectively, when *NHN* is set to be 12. The sensitivity and specificity reach almost the highest value (99.43% and 98.76%, respectively, when the *NHN* is set to be 29). Both of these measures decrease when *NHN* is varied from 80 to 200.

### 3. Individual Diagnostic Rate

The final aim of a lie detection system is to correctly separate the guilty subjects from the innocent subjects. The individual diagnostic rate is the most important evaluation measurement for a lie detection system. As shown in Table 2, the testing accuracies 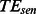 and 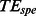 of our proposed method are 99.27% and 98.17%, respectively. Therefore, the averaged testing accuracy is 98.72%, which is the threshold for individual diagnosis (a subject will be classified as a liar if either the sensitivity or the specificity is higher than 98.72%). This number is higher than most results that have been reported in the literature [4], [7], [9] and is also acceptable for practical applications.

## Discussion and Conclusions

In this work, ELM method was first introduced for the purpose of lie detection, and the optimization of the *NHN* of ELM was combined with the F-score feature selection method. As a popular feature selection method, PCA was also combined with ELM, BPNN, and SVM to construct various classification models. The training and testing times, classification accuracy, and network size were used to completely assess the classification performance of these models. Compared with the other classification models tested, the experimental results showed that the proposed method (F-score_ELM) achieves nearly the highest training and testing accuracies for the identification of lying and truth-telling using the most compact network and the shortest training and testing times. Additionally, the proposed method obtains a very high individual diagnostic rate.

The ELM method has many advantages over most other classifiers, such as BPNN and SVM. However, only a few very effective methods have been developed to decide the optimal *NHN*. Most of the investigations focused on the improvement on the ELM algorithm itself. If the feature space is changed, the training procedure needs to be rebooted. Hence, the *NHN* in the ELM should be changed accordingly. We therefore opted to not use the improved ELM algorithms that have been proposed in the past few years and combined ELM with the feature selection method to automatically select the optimal *NHN*. As shown in Table 2, the trained F-score_ELM obtains a smaller *NHN* (*NHN* = 29) with a higher accuracy than the trained ELM model (*NHN = *51). As a result, an advantage of our proposed method is the integration of the optimization of *NHN* and of the feature subset into one optimized procedure. The proposed method provides a new concept for the determination of the optimal *NHN* in ELM algorithms.

With respect to feature selection, the experimental results show that F-score_ELM enhanced the classification performance with the most informative features (*NFS* equals 11) selected by F-score method, compared with the individual ELM model, as shown in Table 3. This experimental result indicates the importance of the feature selection for the ELM classifier.

For the F-score methodology, most researchers always remove redundant features by a threshold calculated based on the *F-score* values. However, this thresholding strategy exhibits some limitations. First, it appears too harsh to directly remove those features with the *F-score* values that are less than the threshold. Second, there should be a close relationship between the *NFS* and the *NHN* of the classifiers. Hence, we abandoned the commonly used technique mentioned above and proposed a combined optimized strategy. It is worth mentioning that the proposed optimization strategy is especially suitable to the ELM classifier because only one classifier parameter in ELM needs to be tuned.

Although we find that *BA_test* of F-score_ELM is slightly lower than that of PCA_ELM (*p* = 0.173>0.05) and significantly lower than that of PCA_SVM (*p* = 0.006<0.01), it was stressed in this study that we evaluated the classification model by its comprehensive performance. Hence the optimal parameter values were not decided only when the training or testing accuracies were maximal. In fact, when *NFS* equals 25, the correspondingly testing accuracy reached its highest value with *BA_test* equal to 99.19%, which is significantly higher (paired *t*-test, *p*<0.01) than that of PCA_ELM (*BA_test = *98.37%). Furthermore, it is noted that there are three inherent limitations in the models that utilize PCA. First, if a new training set is fed into the classification system, the optimal transformed feature set needs to be calculated again because the eigenvector matrix **U**, as mentioned in Feature Selection section, is calculated solely on the input samples. In contrast, the optimal feature subset does not need to be calculated again for the models that utilize F-score methodology. The optimal feature subset with the optimal *NFS* can be directly selected from the new input samples. Second, it is very obvious that F-score has significantly shorter calculation time than PCA. Third, the implication of each feature transformed by PCA is not clear, whereas the features selected by F-score are clear, which could help evaluate the importance level of each original feature. The above discussion appears to indicate that F-score is superior to PCA for the identification of the optimal feature subset.

A good lie detection system with real applications should achieve a high classification accuracy with a lower computational burden. Through the proposed joint optimization, the testing time can be decreased significantly because the smaller *NFS* and *NHN* will be used in testing phase. In addition, in the future, we may need to train on specific subjects to avoid the problem of individual difference. The time required for future training for the proposed method would be also significantly shorter than that for individual ELM and that spent on the originally training procedure because after the original learning procedure, the new training samples with a smaller *NFS* can be fed into the original trained ELM with a corresponding smaller *NHN*. Furthermore, if we found a few important new features, we only need to add these to the originally selected optimal features and then re-perform the above training procedure.

The grid-search strategy was used in many literatures to tune the optimal classifier parameters [40], [46]. In this study, we did not use a more sophisticated algorithm, but employed the grid-search strategy with a SWCV procedure on the feature samples to find the optimal combination of *NHN* and *NFS*. We assumed that this strategy could ransack each combination of *NHN* and *NFS* and hence avoid the local minimum. In addition, because each parameter combination is independent, one can parallelize this searching procedure and thus significantly reduce the learning time of the proposed method.

For the sake of simplicity, we averaged 5 responses to remove the noise in the original ERP signals. We recognized that the averaging method adopted in this study is not very effective for removing noise of P300 because some noise is also time-locked to the stimuli [49]. If more appropriate methods of ERP reconstruction [36], [50] were utilized, our method should be more robust. In addition, other feature selection methods, such as mutual information (MI) and correlation-based method, can be attempted to combine with the ELM to enhance the robustness and the efficiency of the ELM classifier in lie detection or other EEG-based classification problems. This will be the focus of our future works.

Due to space constraints, only two comparable improved ELM algorithms, I-ELM [51] and Pruning ELM (P-ELM) [52], were selected to compare with the proposed method. After training and testing, the following results were obtained: 1) I-ELM: *BA_train = *98.61%, *BA_test = *98.22%, *NHN = *51, *NFS = *31, *TTR = *0.04 hours; 2) P-ELM: *BA_train = *98.63%, *BA_test = *98.18%, *NHN = *50, *NFS = *31, *TTR = *0.21 hours. Based on the principle of these algorithms, it is not surprising that the trained values of *NHN* and *NFS* are nearly the same as the training results of ELM (see Table 2). Using paired *t*-test, we found no significant difference of training and testing accuracies between these two improved algorithms and ELM model (*p* = 0.228>0.01 for I-ELM and *p* = 0.24>0.01 for P-ELM on testing accuracy). As for other improved algorithms such as Kernel based ELM [53] and OS-ELM [54], it may be unsuitable to apply them in this detecting system due to their more complex calculation and training procedure.

The advantage of the proposed method over BAD and BCD is the trials-by-trial analysis, which is particularly beneficial for lie detections. First, we could research the wealth of dynamic variation information between the single trials. Second, based on the testing results on a few trials, the examiner could decide whether to continue to display, terminate or adjust the stimuli content and/or sequence, which would help the examiner make his/her final decision. In contrast, BAD and BCD must make a decision after all the trials are presented. From this perspective, the proposed testing system is based on a small number of stimuli. In fact, the use of many repeated stimuli with little information would induce two problems: fatigue in the subjects and an increase of the countermeasures [7], [9]. This is particular true for real lie detection, when the real criminals are familiar with the stimuli contents, which would result in the criminals resisting the detection procedure on purpose [36]. In our opinion, the development of a lie-detection method with a small number of stimuli is crucial to extend the laboratory study to practical application. In comparison with previously reported PR methods, the presented method reaches a very high classification accuracy with the shortest training and testing times. Therefore, although the classification accuracy in the presented method may be lower than some early reported results, the proposed method achieves a good tradeoff between the various evaluation measures introduced earlier.

Although this study is focused on lie detection, the method reported is not limited to this application. The proposed method has the potential to be used in a variety of classification tasks of brain states, such as identification of ERP or fMRI signals.
